# Phylogeographic Evidence for 2 Genetically Distinct Zoonotic *Plasmodium knowlesi* Parasites, Malaysia

**DOI:** 10.3201/eid2208.151885

**Published:** 2016-08

**Authors:** Ruhani Yusof, Md Atique Ahmed, Jenarun Jelip, Hie Ung Ngian, Sahlawati Mustakim, Hani Mat Hussin, Mun Yik Fong, Rohela Mahmud, Frankie Anak Thomas Sitam, J. Rovie-Ryan Japning, Georges Snounou, Ananias A. Escalante, Yee Ling Lau

**Affiliations:** University of Malaya Faculty of Medicine, Kuala Lumpur, Malaysia (R. Yusof, M.A. Ahmed, M.Y. Fong, R. Mahmud, Y.L. Lau);; Sabah State Health Department, Sabah, Malaysia (J. Jelip);; Hospital Kapit, Sarawak, Malaysia (H.U. Ngian);; Hospital Tengku Microbiology Laboratory, Ampuan Rahimah, Selangor, Malaysia (S. Mustakim);; Kelantan State Health Department, Kelantan, Malaysia (H.M. Hussin);; Department of Wildlife and National Parks Peninsular Malaysia, Kuala Lumpur, Malaysia (F.A.T. Sitam, J. Rovie-Ryan);; Sorbonne Universités, INSERM, Paris, France (G. Snounou);; Temple University, Philadelphia, PA, USA (G. Snounou, A.A. Escalante)

**Keywords:** Cytochrome oxidase 1, simian, human, macaque, parasite, zoonoses, genetic diversity, biological evolution, malaria, *Plasmodium knowlesi*, population genetics, ribosomal RNA, Malaysia, Malaysian Borneo, Peninsular Malaysia, Southeast Asia

## Abstract

Infections of humans with the zoonotic simian malaria parasite *Plasmodium knowlesi* occur throughout Southeast Asia, although most cases have occurred in Malaysia, where *P. knowlesi* is now the dominant malaria species. This apparently skewed distribution prompted an investigation of the phylogeography of this parasite in 2 geographically separated regions of Malaysia, Peninsular Malaysia and Malaysian Borneo. We investigated samples collected from humans and macaques in these regions. Haplotype network analyses of sequences from 2 *P. knowlesi* genes, type A small subunit ribosomal 18S RNA and cytochrome c oxidase subunit I, showed 2 genetically distinct divergent clusters, 1 from each of the 2 regions of Malaysia. We propose that these parasites represent 2 distinct *P. knowlesi* types that independently became zoonotic. These types would have evolved after the sea-level rise at the end of the last ice age, which separated Malaysian Borneo from Peninsular Malaysia.

## Abstract 2

Sequence analyses of genes derived from human and macaque samples led to the proposal that 2 distinct types exist.

The number of malaria cases in Malaysia steadily decreased from a peak of 59,208 in 1995 to 3,850 confirmed cases in 2013; of these, 80% were reported in the 2 states of Malaysian Borneo and the remainder in 6 of the 11 states of Peninsular Malaysia (Figure 1) (*1*). In Malaysia, the simian malarial parasite species *Plasmodium knowlesi* is now the dominant species infecting humans and is >2 times more prevalent than *P. falciparum* or *P. vivax*. Humans were found to be susceptible to *P. knowlesi* when this species was experimentally transmitted to man in 1932, the year in which it was first described (*2*,*3*). In 1965, the first confirmed case of a naturally acquired infection in humans was recorded (*4*). The next naturally acquired confirmed cases were reported in 2004, when a stable focus of *P. knowlesi* was discovered in Sarawak, 1 of 2 states that make up Malaysian Borneo (*5*). Thereafter, transmission of *P. knowlesi* to humans occurred in the second state, Sabah (*6*,*7*), and in neighboring countries (*8*,*9*). 

The natural hosts of *P. knowlesi* are principally the long-tailed (*Macaca fascicularis*) and pig-tailed (*M. nemestrina*) macaques (*10*), 2 species that are widely distributed in the Southeast Asia countries in which cases of *P. knowlesi* have been recorded. To date, human-to-human transmission has not been observed. Infections in humans can cause severe disease that can be fatal (*9**,**11*), underscoring the public health concern raised by this zoonotic simian parasite.

The 2 states of Malaysian Borneo appear to be the epicenter of zoonotic *P*. *knowlesi* infections: 1,391 cases in Malaysian Borneo and 423 cases in Peninsular Malaysia were recorded in 2012. A total of 1,407 PCR-confirmed cases were reported during 2004–2013 in Malaysia, which contrasts with the low number of cases (n = 136) reported from neighboring countries (*9*): Cambodia (n = 1), China (n = 36), Indonesia (n = 1), Myanmar (n = 14), the Philippines (n = 5), Singapore (n = 2), Thailand (n = 36), and Vietnam (n = 32). The reasons for this uneven distribution remain unclear. Geographic variation in mosquito species and human social factors could be an explanation; it is also possible that the parasite populations circulating on the island of Borneo are distinct from those found in continental Malaysia. The *P. knowlesi* strains that had been studied in earlier years displayed distinct biologic characteristics, which in some cases led malariologists to propose distinct *P. knowlesi* subspecies (*12*). Such differences could indicate that local ecologic factors are influential and that each *P. knowlesi* subspecies became a zoonosis independently in each geographic area.

To explore whether the *P. knowlesi* populations in Malaysia differed and independently became zoonoses, we focused on 2 genes that have been extensively used for phylogenetic studies (*13**–**16*): 1 nuclear, encoding the type A small subunit ribosomal 18S RNA (*PkA-type 18S rRNA*), and 1 mitochondrial, encoding the cytochrome oxidase subunit I protein (*PkCOX1*). Using samples collected from humans and macaques in both regions of the country, we generated the relevant sequences, compared them to those published previously (*17*), and conducted phylogenetic and population genetic analyses.

## Materials and Methods

### Sample Collection

The Medical Research Ethic Committee of the Ministry of Health Malaysia, Sabah State Director of Health, Kelantan State Director of Health, and directors of state and district hospitals approved this study. Ethical approval was granted by the Medical Research and Ethics Committee of the Malaysian Ministry of Health (Reference Number: KKM/NIHSEC/800/-2/2/2/P13–316), the Medical Ethics Committee of University Malaya Medical Centre, and the Department of Wildlife and National Parks.

**Figure 1:**
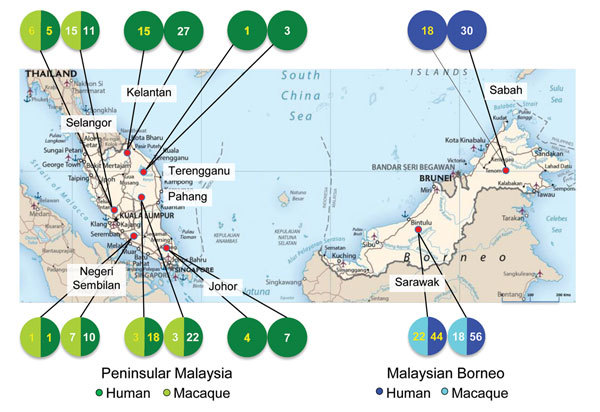
Geographic origin of the genetic sequences generated during study of *Plasmodium knowlesi* parasite populations, Malaysia. The numbers in each circle refer to the number of sequences (macaque or human) obtained for the genes *P.* *knowlesi* type A small subunit ribosomal 18S RNA (numbers in white) and *P.* *knowlesi* cytochrome oxidase subunit I (numbers in yellow).

We used previously collected human blood samples for this study (*7*): 78 microscopically confirmed *P. knowlesi*–positive blood samples from patients in 8 states in Malaysia, including Sabah and Sarawak in Malaysian Borneo (Figure 1). We also examined blood samples from 8 long-tailed macaques collected during routine surveys by the Department of Wildlife and National Parks in the Peninsular Malaysia states of Pahang, Selangor, and Negeri Sembilan. All samples were collected during September 2012–December 2013 (Tables 1, 2). In addition to these samples, we included previously published sequences deposited into GenBank during 2003–2015 in the analyses; these sequences were derived from samples collected from humans and macaques (Technical Appendix Table).

**Table 1:** Samples and sequences obtained for the *PkA-type 18S rRNA* gene used to distinguish 2 distinct *Plasmodium* *knowlesi* parasite populations in Malaysia*

Population location				No. sequences						Total sequences obtained
	No. samples†			Human			Macaque			
	Human	Macaque		This study‡	GenBank§		This study‡	GenBank§		
Peninsular Malaysia										
Kelantan	13	ND		27	ND		ND	ND		27
Johor	3	ND		7	ND		ND	ND		7
Selangor	7	5		11	ND		15	ND		26
Terengganu	2	ND		3	ND		ND	ND		3
Pahang	14	1		21	1		3	ND		25
Negeri Sembilan	4	2		10			6	1		17
Malaysian Borneo										
Sarawak	22	ND		48	8		ND	18		74
Sabah	13	ND		30	ND		ND			30
Total samples	86			78	8		8	6		86 human + 14 macaque
Total sequences	NA			157	9		24	19		166 human + 43 macaque

*NA, not applicable; ND, no data; *PkA-type 18S rRNA*, *P.* *knowlesi* type A small subunit ribosomal 18S RNA. †Previously collected samples used for this study. ‡GenBank accession nos. KT852845–KT852938 and KJ917815–KJ917903. §GenBank accession numbers listed in online Technical Appendix Table (http://wwwnc.cdc.gov/EID/article/22/8/15-1885-Techapp1.pdf).

**Table 2:** Samples and sequences obtained for the *PkCOX1* gene used to distinguish the 2 distinct *Plasmodium* *knowlesi* parasite populations in Malaysia*

Location				No. sequences					Total sequences obtained
	No. samples†			Human			Macaque		
	Human	Macaque		This study‡	GenBank§		This study‡	GenBank§	
Peninsular Malaysia									
Kelantan	13	ND		15	ND		ND	ND	15
Johor	3	ND		4	ND		ND	ND	4
Selangor	7	5		5	ND		5	1	11
Terengganu	2	ND		1	ND		0	ND	1
Pahang	14	1		18	ND		2	1	21
Negeri Sembilan	4	2		ND	1		1	ND	2
Malaysian Borneo									
Sarawak	22	ND		23	21		ND	22	66
Sabah	13	ND		18	ND		ND	ND	18
Total samples	NA	86		78	22		8	12	100 human + 20 macaque
Total sequences	NA	NA		84	22		8	24	106 human + 32 macaque

*NA, not applicable; ND, no data; *PkCOX1* *P.* *knowlesi* cytochrome oxidase subunit I. †Previously collected samples used for this study. ‡GenBank accession nos.KT900705–KT900797). §GenBank accession numbers listed in online Technical Appendix Table (http://wwwnc.cdc.gov/EID/article/22/8/15-1885-Techapp1.pdf).

### Amplification and Sequencing of Gene Fragments

Genomic DNA was extracted from the human and macaque blood samples by using the DNeasy Blood Tissue Kit (QIAGEN, Hilden, Germany), according to the manufacturers’ protocol. An established nested PCR protocol was used to test the samples; all tested positive for *P. knowlesi* only (*5*). 

The *PkA-type 18S rRNA* and *PkCOX1* genes were then amplified (MyCycler, Bio-Rad, Hercules, CA). In a primary amplification reaction, a *PkA-type 18S rRNA* fragment of 1.1. kb was obtained by using the oligonucleotide primer pair rPLU5+rPLU6 (*18*). The amplification reaction was completed in a mixture containing 1X Green Go Taq Flexi Buffer (Promega, Madison, WI, USA); 4.0 mol/Lmagnesium chloride solution; 0.2 mol/L dNTP Mix (Promega), 0.2 μM of each primer; 1 U GoTaq Flexi DNA Polymerase (Promega); and 4 μL of DNA template combined with nuclease–free water to obtain a final volume of 25 μL. The PCR amplification was initiated at 95°C for 10 min, then by 35 cycles of denaturation at 94°C for 30 s, annealing at 55°C for 30 s, extension at 72°C for 1 min, and a final extension at 72°C for 5 min. For the secondary amplification, 4 μL of the primary amplification product were used as a template by using forward primer Pkl1 (5′-ACATAACTGATGCCTCCGCGTA) and reverse primer Pkl2 (5′-CACACATCGTTCCTCTAAGAAGC) to obtain a 986–990-bp fragment. The reaction mixture and the cycling conditions were as above with minor modification for the 35 cycles: denaturation at 94°C for 1 min, annealing at 53°C for 1 min, extension at 72°C for 1 min, and final extension at 72°C for 10 min. The amplified PCR fragments were cloned into the pGEM-T Vector (Invitrogen, Carlsbad, CA, USA). Plasmids purified from >2 positive clones from each ligation mixture were selected for sequencing (First Base Laboratories Sdn Bhd, Malaysia). Any polymorphism that was not observed in >2 samples was only included in the analysis if its validity was confirmed by a repeated cycle of amplification, cloning, and sequencing.

For the *PkCOX1* gene, amplification was achieved by using forward (5′-GCCAGGATTATTTGGAGG) and reverse (5′-CAGGAATACGTCTAGGCA) primers to obtain a 1,116-bp fragment. These primers were designed based on a published gene sequence (GenBank accession no. AY598141). The amplification reaction was achieved as above. The PCR amplification was initiated at 95°C for 3 min, then denatured for 35 cycles at 94°C for 1 min, annealed at 52°C for 1 min, extended at 72°C for 1 minute, and put through final extension at 72°C for 10 min. The purified amplified fragments were then sent for sequencing.

### Sequence Editing and Alignment

We analyzed the DNA sequences using BioEdit Sequence Alignment Editor Software (http://www.mbio.ncsu.edu/BioEdit/bioedit.html) on the reference *P. knowlesi* H-strain (GenBank accession no. AM910985) for the *PkA-type 18S rRNA* and the *P. knowlesi* mitochondrial sequence (GenBank accession no. NC 00723244) for the *PkCOX1* gene. Results were exported to MEGA 5.6 software (http://www.megasoftware.net) for further alignment and analysis. We performed similarity searches using BLAST (http://blast.ncbi.nlm.nih.gov/Blast.cgi). We obtained 28 additional *PkA-type 18S RNA* sequences derived from *P. knowlesi*–infected samples (9 from humans and 19 from macaques) and 46 additional *PkCOX1* sequences derived from *P. knowlesi*-infected samples (22 from humans and 24 from macaques) from GenBank and included these sequences in the analysis (online Technical Appendix Table).

### Haplotype Network Analysis

We estimated polymorphism of the *PkA-type 18S rRNA* and *PkCOX1* genes by computing haplotype diversity (Hd), number of haplotypes (h), nucleotide diversity (π), number of polymorphic sites, and the average number of pairwise nucleotide differences using DnaSP version 5.10.01 software (Bio­Soft http://en.bio-soft.net/). We constructed haplotype networks for *PkA-type 18S rRNA* and *PkCOX1* genes based on their polymorphic sites by using the median-joining method in NETWORK version 4.6.1.2 software (Fluxus Technology Ltd, Suffolk, UK). We inferred the genealogical haplotype network using the sequences of *P. knowlesi* human and macaque isolates from Peninsular Malaysia and Malaysian Borneo. Where available, we included sequences from the *P. knowlesi* H and Nuri strains as references.

### Population Genetic Structure Analysis

To define genetic structure of the *P. knowlesi* parasite population in Malaysia, we used STRUCTURE version 2.3.4 software (The Pritchard Lab, Stanford University, Stanford, CA, USA) that deploys the Bayesian model–based clustering approach. We estimated the most probable number of populations (K) using an admixture model. All sample data (for both genes) were run for values K = 1–8, each with a total of 15 iterations. We used 500,000 Markov Chain Monte Carlo generations for each run after a burn-in of 50,000 steps. The most likely number K in the data was estimated by calculating ΔK values and identifying the K value that maximizes the log probability of data, lnP(D) (*19*). The most probable K value was then calculated according to Evanno’s method (*20*) by using the webpage interface STRUCTURE Harvester (*21*). We also used ARLEQUIN version 3.5.1.3 software (University of Berne, Berne, Switzerland) to compute pairwise differences (*F_ST_*) between populations (i.e., humans and macaques from Peninsular Malaysia and Malaysian Borneo) (*22*) from haplotypes that showed 10,100 permutations. *F_ST_* is a comparison of the sum of genetic variability within and between populations on the basis of the differences in allelic frequencies. We interpreted *F_ST_* values as no (0), low (>0–0.05), moderate (0.05–0.15), and high (0.15–0.25) genetic differentiation.

### Neutrality and Demographic Analysis

We examined departure from a strict neutral model, including demographic expansions, on the basis of pairwise mismatch distribution, the Tajima D test (*23*), Fu and Li D (*24*), Fu and Li F, and Fu Fs statistics (*25*) using DnaSP version. 5.10.01 software (*26*). Significant negative values for these tests indicate either a purifying selection or population expansion, and positive values indicate balancing selection.

## Results

The sequences analyzed in this study were derived from 130 *P. knowlesi*–infected blood samples obtained from 23 macaques and 107 humans. We analyzed a total of 209 *PkA-type 18S RNA* sequences (105 from Peninsular Malaysia and 104 from Malaysian Borneo) and 138 *PkCOX1* sequences (54 from Peninsular Malaysia and 84 from Malaysian Borneo). 

### Gene Diversity Indices

Analysis of the molecular polymorphism within the 209 partial *PkA-type 18S rRNA* sequences (945 bp) revealed moderately polymorphic sequences (π = 0.00324 ± 0.00019). Overall, 137 polymorphic sites yielded 93 haplotypes. Nucleotide and haplotype diversities were broadly similar for both Peninsular Malaysia and Malaysian Borneo samples (Table 3). Single-nucleotide polymorphisms were scattered throughout the gene; most the Peninsular Malaysia sequences displayed a distinct single nucleotide polymorphism (G→A at position 830) (Technical Appendix Table).

**Table 3:** Genetic characteristics of the 2 genetically distinct zoonotic *Plasmodium knowlesi* parasite populations in Malaysia*

Gene and location	No. samples	No. haplotypes	No. polymorphic sites	Haplotype diversity ±SD	Nucleotide diversity ±SD	Average. no. nucleotide differences
*PkA-type 18S rRNA*						
Peninsular Malaysia	105	47	62	0.906 ±0.0.025	0. 0.00284 ±0.00024	2.66667
Malaysian Borneo	105	48	85	0.871 ±0.031	0.00276 ±0.0.00029	2.59341
Overall	210	93	137	0.941 ±0.0.011	0.00324 ±0.00019	3.03550
*PkCOX1*						
Peninsular Malaysia	53	20	20	0.827 ±0.039	0.00141 ±0.00018	1.52685
Malaysian Borneo	84	25	25	0.676 ±0.057	0.00115 ±0.00014	1.24498
Overall	137	44	39	0.848 ±0.025	0.00215 ±0.00013	2.33104

*PkA-type 18S rRNA, *P.* *knowlesi* type A small subunit ribosomal 18S RNA;* PkCOX1* *P.* *knowlesi* cytochrome oxidase subunit I.

Analysis of the molecular polymorphism within the 138 partial *P. knowlesi* mitochondrial *COX1* sequences (1,082 bp) revealed low instances of polymorphism (π = 0.00215 + 0.00013). Overall, 61 polymorphic sites yielded 44 haplotypes. Although nucleotide diversities were similar for sequences from both regions, haplotype diversity was higher for the sequences from Peninsular Malaysia (h = 19, Hd = 0.827 ± 0.039) than for those from Malaysian Borneo (h = 25, Hd = 0.676 ± 0.057) (Table 3). Sequences from Malaysian Borneo were distinguished from those from Peninsular Malaysia by 2 distinct single nucleotide polymorphisms (G→A at position 166 andT→C at position 659) (Technical Appendix Figure).

### Haplotype network

**Figure 2:**
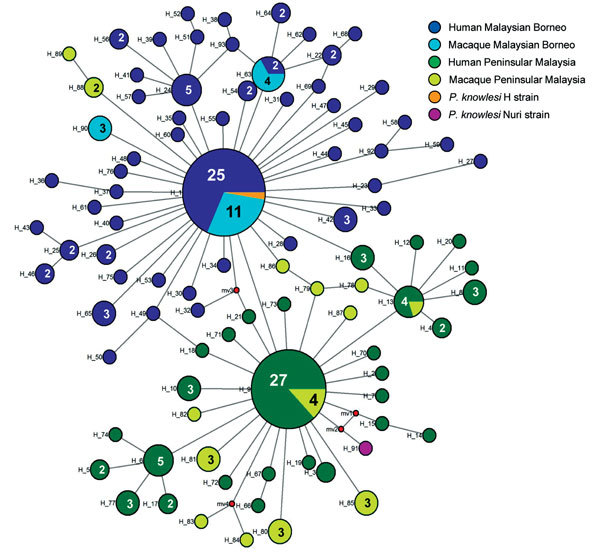
Median-joining networks of *Plasmodium* *knowlesi* type A small subunit ribosomal 18S RNA haplotypes from Malaysia. The genealogical haplotype network shows the relationships among the 93 haplotypes present in the 209 sequences obtained from human and macaque samples from Peninsular Malaysia and Malaysian Borneo. Each distinct haplotype has been designated a number (H_n). Circle sizes represent the frequencies of the corresponding haplotype (the number is indicated for those that were observed >1×). Small red nodes are hypothetical median vectors created by the program to connect sampled haplotypes into a parsimonious network. Distances between nodes are arbitrary.

**Figure 3:**
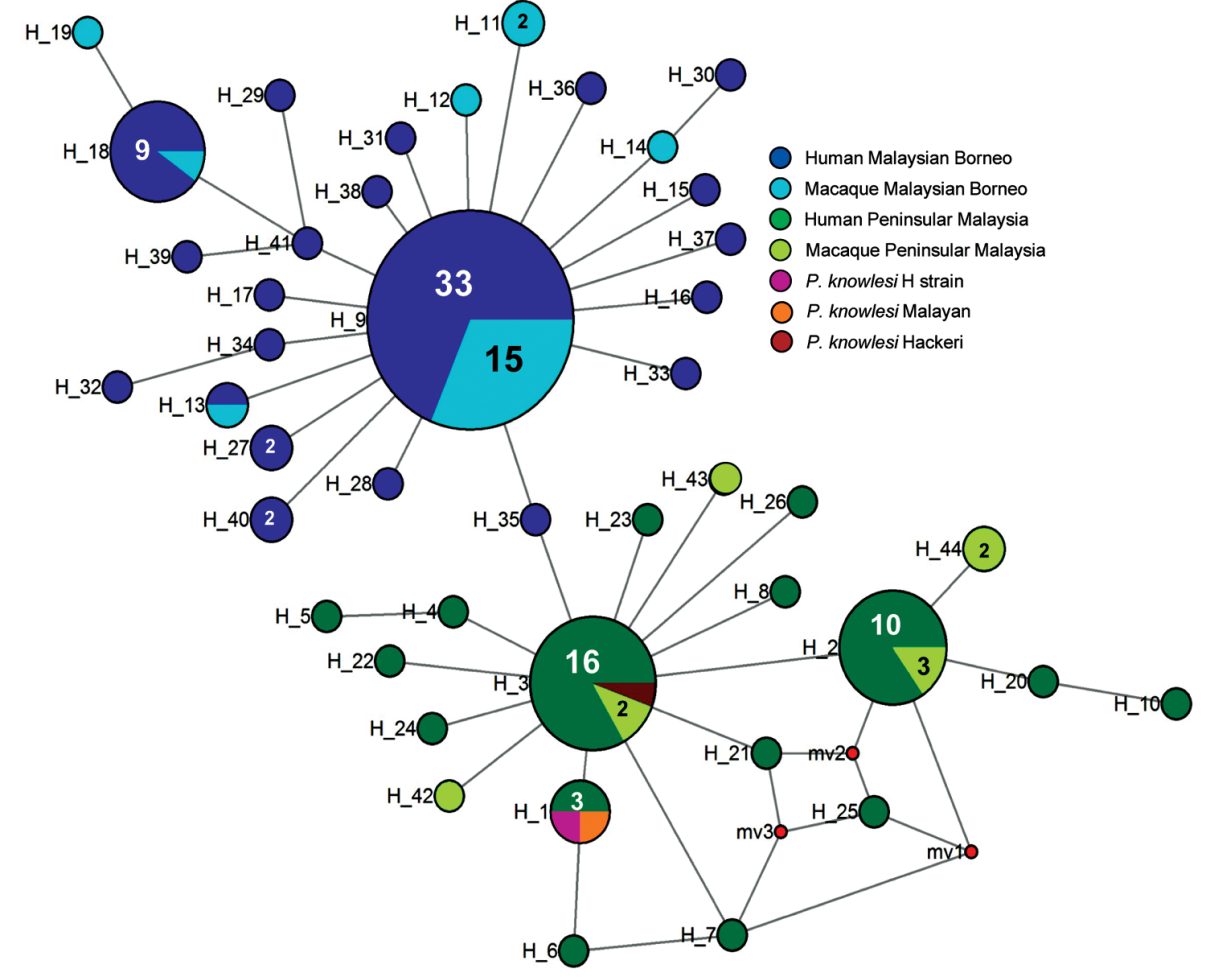
Median-joining networks of *Plasmodium* *knowlesi* cytochrome oxidase subunit I haplotypes from Malaysia. The genealogical haplotype network shows the relationships among the 44 haplotypes present in the 138 sequences obtained from human and macaque samples from Peninsular Malaysia and Malaysian Borneo. Each distinct haplotype has been designated a number (H_n). Circle sizes represents the frequencies of the corresponding haplotype (the number is indicated for those that were observed more than once). Small red nodes are hypothetical median vectors created by the program to connect sampled haplotypes into a parsimonious network. Distances between nodes are arbitrary.

DNA sequence variation in our phylogeographic study is more clearly observed in a haplotype network. The network tree for the *PkA-type 18S RNA* haplotypes (Figure 2) showed 2 distinct *P. knowlesi* populations that, with 1 exception, clustered exclusively to 1 of the 2 regions of Malaysia: Peninsular Malaysia (n = 47) and Malaysian Borneo (n = 48). The exception was of 2 haplotypes derived from 1 macaque sample (haplotypes 88 and 89) from Peninsular Malaysia that clustered with the Malaysian Borneo haplotypes. In each cluster, only 2 haplotypes were shared by humans and macaques, but in each case 1 was dominant (in Malaysian Borneo, haplotype 1: n = 38, human = 27, macaque = 11; and in Peninsular Malaysia, haplotype 9: n = 31, human = 27, macaque = 4). The network tree for the *PkCOX1* genes showed a similar pattern to that of *PkA-type 18S rRNA*: it had 2 geographically distinct *P. knowlesi* populations (n = 19 for Peninsular Malaysia and n = 25 for Malaysian Borneo) (Figure 3), and dominant haplotypes in each cluster were shared between humans and macaques (Figure 3).

**Figure 4:**
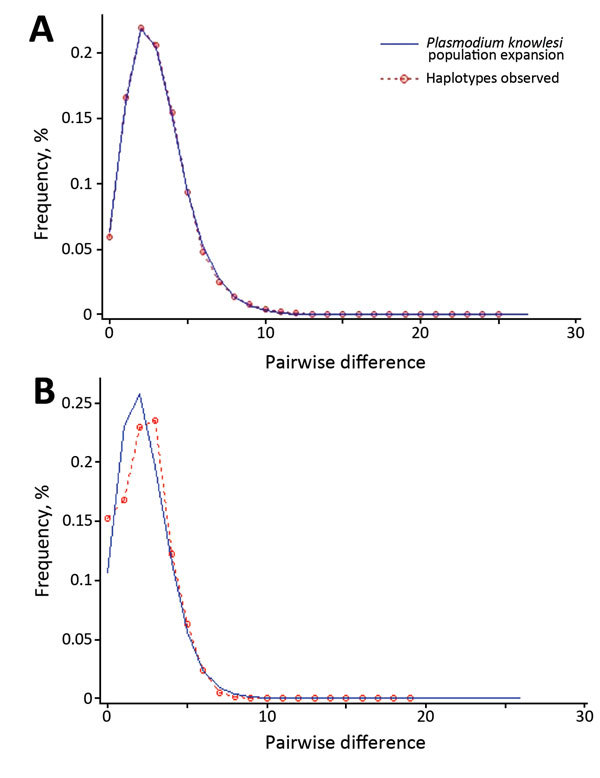
Pairwise mismatch distribution of *Plasmodium knowlesi* parasite populations, Malaysia. A) Type A small subunit ribosomal 18S RNA; B) cytochrome oxidase subunit I. Red dotted lines represent the observed frequencies of the pairwise differences among mitochondrial DNA sequences; blue lines represent the expected curve for a population that has undergone a demographic expansion.

The excess of unique *PkA-type 18S rRNA* and *PkCOX1* haplotypes observed for the *P. knowlesi* populations in humans (Figures 2, 3) is indicative of an evolutionarily recent population expansion. A signature of population expansion was also evident from the unimodal shape of the pairwise mismatch distribution of the *PkA-type 18S rRNA* and *PkCOX1* genes (Figure 4). Calculations by using Tajima D, Fu and Li D and F, and Fu Fs statistics also showed significant negative values (p = 0.05–0.001; Table 4). However, the low number of samples and consequent sequences from macaques precludes any meaningful comparison.

**Table 4:** Results of statistical testing for neutrality of *Plasmodium knowlesi* parasite populations in Malaysia

Gene and location	Tajima D (*23*)	Fu and Li D (*24*)	Fu and Li F (*25*)	Fu Fs (*26*)
*PkA-type 18S rRNA*				
Peninsular Malaysia	–2.49764*	–3.71579 †	–3.85230 †	–56.133
Malaysian Borneo	–2.73586*	–6.47796 †	–5.87592 †	–59.698
Overall	–2.71801*	–7.08484†	–5.97285†	–151.882
*PkCOX1*				
Peninsular Malaysia	–2.06554‡	–3.20519‡	–3.33283†	–18.092
Malaysian Borneo	–2.28662†	–3.60846†	–3.71725†	–27.990
Overall	–2.02607‡	–4.40748†	–4.11218†	–48.131

*PkA-type 18S rRNA, *P.* *knowlesi* type A small subunit ribosomal 18S RNA;* PkCOX1* *P.* *knowlesi* cytochrome oxidase subunit I. *p<0.001.  †p<0.02. ‡p<0.05.

### Population Structure

**Figure 5:**
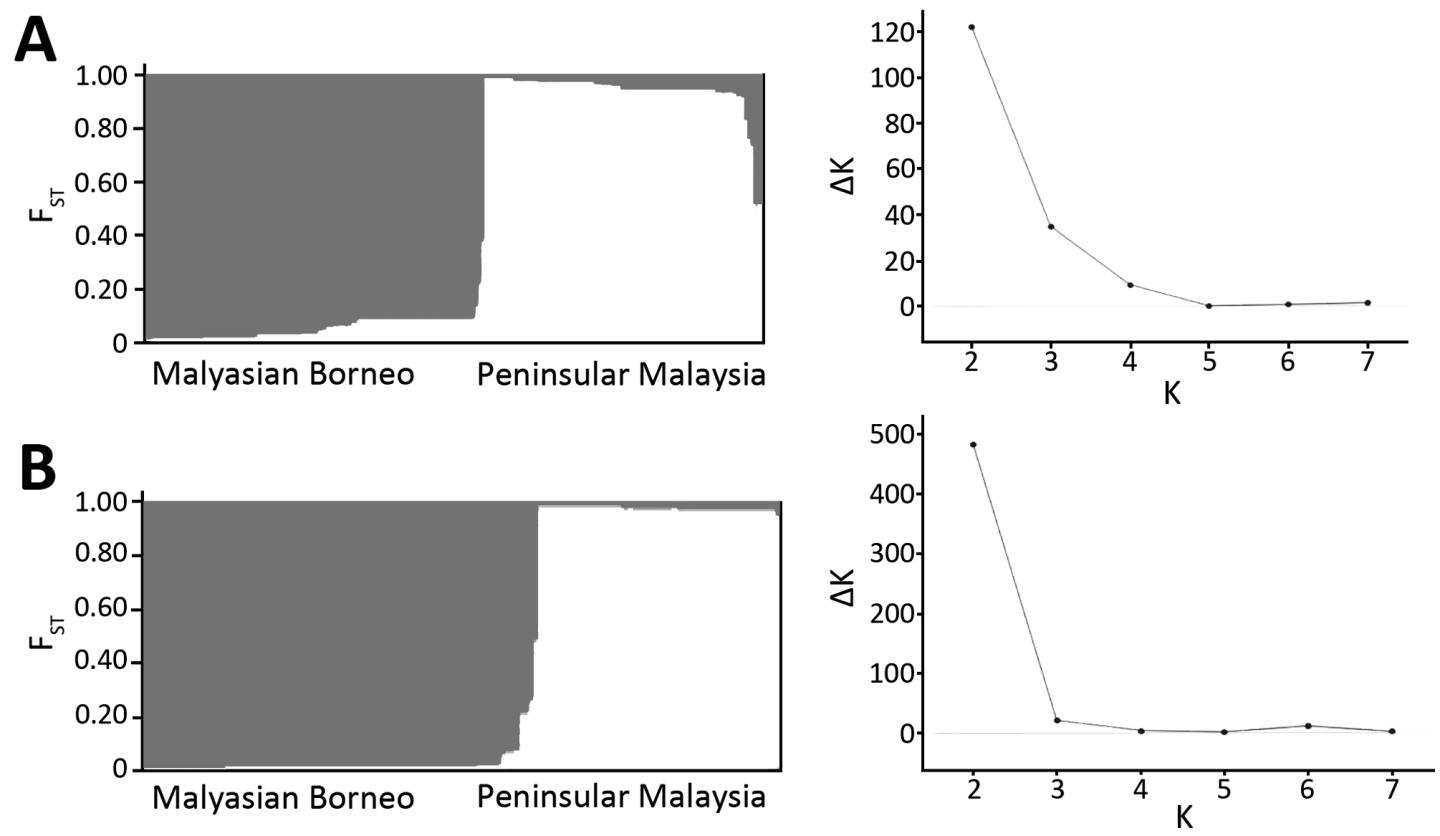
Most likely number of *Plasmodium knowlesi* parasite subpopulation haplotype clusters (K) in Malaysian Borneo (gray) and Peninsular Malaysia (white). A) Type A small subunit ribosomal 18S RNA (K = 2, ΔK = 121.79), including comparison of K and ΔK; B) cytochrome oxidase subunit I (K = 2, ΔK = 481.27), including comparison between K and ΔK. Relationships were determined by using Bayesian model–based STRUCTURE version 2.3.4 software (The Pritchard Laboratory, Stanford University, Stanford, CA, SUA). ΔK = mean (|L′′(K)|)/sd(L(K)). Vertical axes represent membership coefficient values.

We used a Bayesian admixture model implemented in STRUCTURE to calculate the potential number of *P. knowlesi* parasite populations within Malaysia. Because the study samples were collected from 8 different states of Malaysia (Figure 1), we used K values from 1 to 8 for the analysis. For both genes, significant genetic structure was found between the parasite populations when K = 2 (Figure 5), indicating 2 distinct populations clustered to 1 of the 2 main regions of Malaysia (*PkA-type 18S rRNA*, K = 2, ΔK = 121.79; *PkCOX1*, K = 2, ΔK = 481.27). In *PkA-type 18S rRNA* and *PkCOX1* sequences, we also estimated pairwise *F_ST_* values using ARLEQUIN software to determine to what extent population differentiation exists within *P. knowlesi* in Malaysia on the basis of host and geographic origin, i.e., between humans and macaques and between Peninsular Malaysia and Malaysian Borneo. This analysis revealed particularly high population differentiation *F_ST_* values (>0.21 for *PkA-type 18S rRNA* and >0.60 for *PkCOX1*) for samples originating from Peninsular Malaysia and Malaysian Borneo irrespective of the host (human or macaque) from which they were collected (Table 5). For the macaque and human population within the same geographic region, the *F_ST_* values were low (<0.05) (Table 5), suggesting that parasitic transmission was confined to each of the regions. These results are concordant with the haplotype network analysis.

**Table 5:** *F_ST _*results for pairwise population comparisons of 2 genetically distinct zoonotic *Plasmodium knowlesi* parasite populations and associated significance, Malaysia*†

Gene and location	Haplotype	*F_ST _*values				
		HuPen	MaPen	HuBor	MaBor	
*PkA-type 18S rRNA*						
Peninsular Malaysia	HuPen	NA	‡	‡	‡	
	MaPen	0.0483	NA	‡	‡	
Malaysian Borneo	HuBor	0.2710	0.2167	NA	NS	
	MaBor	0.3134	0.3090	0.0008	NA	
*PkCOX1*						
Peninsular Malaysia	HuPen	NA	§	‡	‡	
	MaPen	0.0461	NA	‡	‡	
Malaysian Borneo	HuBor	0.6008	0.6303	NA	NS	
	MaBor	0.6219	0.6925	0.0007	NA	

*HuBor, human haplotypes from Malaysian Borneo; HuPen, human haplotypes from Peninsular Malaysia; MaBor, Macaque haplotypes from Malaysian Borneo; MaPen, macaque haplotype from Peninsular Malaysia; NA, not applicable; NS, not significant; *PkA-type 18S rRNA*, *P.* *knowlesi* type A small subunit ribosomal 18S RNA;* PkCOX1* *P.* *knowlesi* cytochrome oxidase subunit I. †ARLEQUIN (University of Berne, Berne, Switzerland) software package version 3.5.1.3 was used to compute pairwise differences between populations (i.e., humans and macaques from Peninsular Malaysia and Malaysian Borneo). ‡p<0.001; p values computed with 10,100 permutations. §p<0.05.

## Discussion

The results of the various analyses conducted on the *P. knowlesi* parasites collected from Peninsular Malaysia and Malaysian Borneo strongly support the conclusion that the 2 geographically separated regions of this country harbor genetically distinct *P. knowlesi* populations. Haplotype diversity, a measure of species evenness (low values indicate skewing toward a few predominant haplotypes), was high for both the Peninsular Malaysia (0.906 ± 0. 0.025) and the Malaysian Borneo (0.871 ± 0.031) isolates, which may indicate a sustained transmission of *P. knowlesi* in both regions of Malaysia over long periods. Similar high haplotype diversity values have been reported for the *P. knowlesi csp* gene in isolates from Sarawak (*17*). Nucleotide diversity, however, was low for the genes analyzed in this study, irrespective of the samples’ geographic or host origins, indicating that only minor differences occurred between the haplotypes observed (Technical Appendix Table, Figure). A similar pattern has been observed for geographically separated *P. vivax* populations between which gene flow is limited (*14*).

The relatively large sample group size and the short genetic distances between the intraspecific sequences in this study are not suited for phylogenetic studies that aim to reconstruct genealogies. Therefore, we subjected the sequences to a median-joining haplotype network analysis, which showed that the network consisted mostly of unique haplotypes that clearly form 2 clusters: 1 comprised the samples obtained from Peninsular Malaysia, and the other the samples from Malaysian Borneo (Figures 2, 3). Within each cluster, the dominant haplotypes were shared between humans and macaques; a similar observation was previously reported for a sample set collected in Sarawak (*17*). Additional population structure analyses showed very high genetic differentiation between 2 distinct *P. knowlesi* populations from the 2 geographic regions and very low genetic differentiation between the human and macaque parasites within each of these regions (Figure 5). These observations strongly support the conclusion that humans are susceptible to infection by any of the *P. knowlesi* types circulating in macaques.

The question arises as to how these 2 distinct populations arose. Analyses of the complete mitochondrial DNA revealed that *P. knowlesi* parasites were present in macaques around 65,000 years ago, before human settlement in Southeast Asia (*17*). Although DNA sequences isolated in our study are too short to perform a comparable analysis, it has been proposed that the macaque populations of Borneo became isolated from those of Peninsular Malaysia, Java, and Sumatra around 15,000 years ago, when the rise in the level of the South China Sea at the end of the last ice age submerged parts of Sundaland (*27*). Thus, *P. knowlesi* populations likely became isolated, along with their natural vertebrate and insect hosts, and consequently evolved separately. This mechanism of geographic isolation and host demography is considered important in the differentiation and origin of *P. knowlesi* parasites and other closely related *Plasmodium* species, for which diversity is probably underestimated (*16*,*28*). We propose that the 2 distinct *P. knowlesi* populations currently circulating in Peninsular Malaysia and Malaysian of Borneo correspond to 2 independently evolving populations. The *PkCOX1* haplotypes available for the *P. knowlesi* isolates from Peninsular Malaysia (Malayan, GenBank accession no. AB444106.1; Hackeri, accession no. AB444107.1; H, accession no. AB444108) fall within the Peninsular Malaysia cluster; corresponding *PkA-type 18S rRNA* sequences are not available. 

The 3 *P. knowlesi* subspecies proposed to date (*12*) were found in distinct locations (Taiwan, Java, and Peninsular Malaysia). These parasites were not studied further and confirmation of their subspecific status remains to be confirmed, but no material suitable for genetic analysis is available. We note that evidence of potential hybrid forms was obtained from a single sample from a macaque from Sabah and for the H strain. The low frequency of such forms suggests a recent admixture through increased human movement between the 2 geographic regions of Malaysia, biologic factors that limit their fitness, or both. The clear geographic genetic differentiation and the indiscriminate distribution between human and macaque hosts for the parasites within each cluster strongly indicate that *P. knowlesi* became zoonotic independently in the 2 regions. Given that *P. knowlesi* has long been zoonotic, the data also indicate that human-to-human transmission has not been established over time.

While our study was underway, *P. knowlesi* populations from Sarawak were shown to be dimorphic across their genome (*29*,*30*). Furthermore, analyses based on microsatellite diversity identified 2 distinct parasite groups across the whole of Malaysia that strongly cluster with the macaque host species but not in the human samples: 1 was dominant in *M. fascicularis* macaques and the other in *M. nemestrina* macaques (*31*). These distinct groups do not correspond to the 2 distinct clusters we propose because their pattern of distribution is incongruent with the marked geographic distribution we report. It is likely that they represent a more recent population structure driven by adaptation to the macaque as vector and host. Considering the differences in the mutation rates between mitochondrial and nuclear genes and microsatellites (*32**,**33*), the data from the microsatellite analyses would be expected to detect recent population structuring, and our data from the nuclear and mitochondrial genes would reveal a more ancient geographic isolation.

In conclusion, it is clear that the *P. knowlesi* malaria parasites are not a homogeneous group but form a complex of related types, possibly including emerging subspecies, of which evolution and distribution has been shaped by past and recent events. It is equally clear that further analyses encompassing a larger number of genetic loci or whole genomes from sample sets collected in Malaysia, as well as in neighboring countries, will be needed to obtain a more comprehensive picture of the phylogeographic distribution and population structure of *P. knowlesi*. The zoonotic potential of these parasites constitutes a threat to the efforts to eliminate malaria in Southeast Asia. Thus, more epidemiologic and biologic investigations are necessary to help devise strategies that will minimize if not eliminate this threat.

Technical Appendix Accession numbers of sequences retrieved from GenBank database included in analyses of phylogeographic evidence for 2 genetically distinct zoonotic *Plasmodium knowlesi* parasites and nucleotide polymorphism in the cytochrome oxidase 1 (*cox1*) gene of *P. knowlesi* isolates from humans and macaques in Malaysia.
