# Supplementary material for: Phylogeographic Evidence for 2 Genetically Distinct Zoonotic Plasmodium knowlesi Parasites, Malaysia
Source: Emerg Infect Dis. 2016 Aug;22(8):1371–80. doi: 10.3201/eid2208.151885 (PMC4982179; doi:10.3201/eid2208.151885)
Supplement: Technical Appendix — Accession numbers of sequences retrieved from GenBank database included in analyses of phylogeographic evidence for 2 genetically distinct zoonotic Plasmodium knowlesi parasites and nucleotide polymorphism in the cytochrome oxidase 1 (cox1) gene of P. knowlesi isolates from humans and macaques in Malaysia. [file 15-1885-Techapp-s1.pdf]

Article DOI: http://dx.doi.org/10.3201/eid2208.151885

Phylogeographic Evidence for 2 Genetically Distinct Zoonotic *Plasmodium knowlesi* Parasites, Malaysia

Technical Appendix

Technical Appendix Table. Accession numbers of sequences retrieved from GenBank database included in analyses of phylogeographic evidence for two genetically distinct zoonotic *Plasmodium knowlesi* parasites in Malaysia

	PkA-type 18S rRNA		*PkCOX1*	
	Source		Source	
Location	Humans	Macaques	Humans	Macaques
Malaysian Borneo				
	7 Samples	5 Samples	21 Samples	10 Samples
	KH33_(AY327549)	A2_(DQ350264)	KH45H1_(EU880446.1)	LT3H20_(EU880472.1)
	KH35_(AY327550)	LT3-B6_(DQ641524)	KH54H4_(EU880447.1)	LT4H5_(EU880474.1)
	KH35_(AY327552)	LT3-C7_(DQ350266)	KH58H2_(EU880448.1)	LT15H21_(EU880475.1)
	KH43_(AY327551)	LT4-A1_(DQ350267)	KH100H1_(EU880449.1)	LT15H22_(EU880476.1)
	KH96_(AY327553)	LT4-A7_(DQ350268)	KH108H12_(EU880451.1)	LT20H5_(EU880479.1)
	KH107_(AY327554	LT4-B1_(DQ350269)	KH137H15_(EU880452.1)	LT20H23_(EU880480.1)
	KH115_(AY327555)	LT4-C18_(FJ619069.1)	KH176H2_(EU880454.1)	LT20H24_(EU880481.1)
	KH131_(AY327556)	LT4-C20_(DQ641525)	KH185H3_(EU880455.1)	LT20H25_(EU880482.1)
	NA	LT20-C1_(FJ619088)	KH225H11_(EU880457.1)	LT26H26_(EU880483.1)
	NA	LT20-C5_(FJ619090)	KH229H4_(EU880458.1)	LT26H27_(EU880484.1)
	NA	LT22-A1_(DQ641518)	KH273H19_(EU880459.1)	LT48H6_(EU880485.1)
	NA	LT22-A17_(DQ641519)	KH275H13_(EU880460.1)	LT48H28_(EU880486.1)
	NA	LT22-B2_(DQ641520)	KH294H10_(EU880461.1)	LT51H6_(EU880487.1)
	NA	LT22-B18_(DQ641521)	KH343H18_(EU880462.1)	LT51H29_(EU880488.1)
	NA	LT22-C4_(DQ641522)	KH369H14_(EU880463.1)	LT53H30_(EU880489.1)
	NA	LT22-C7_(DQ641523)	KH381H3_(EU880465.1)	LT53H31_(EU880490.1)
	NA	LT33-D1_(FJ619097)	KH396H16_(EU880466.1)	LT53H32_(EU880491.1)
	NA	LT33-D12_(FJ619098	KH397H8_(EU880464.1)	LT54H33_(EU880493.1)
	NA	NA	KH431H9_(EU880467.1)	LT57H34_(EU880495.1)
	NA	NA	KH433H7_(EU880468.1)	LT57H35_(EU880496.1)
	NA	NA	KH468H17_(EU880470.1)	LT57H36_(EU880497.1)
	NA	NA	NA	LT57H37_(EU880498.1)
Peninsular Malaysia				
	AM910985 (Pk H strain)	AY327557 (Pk Nuri)	AB444108 (Pk H strain)	AB444107.1 (Pk Hackeri)
	NA	NA	NA	AB444106.1 (Pk Malayan)

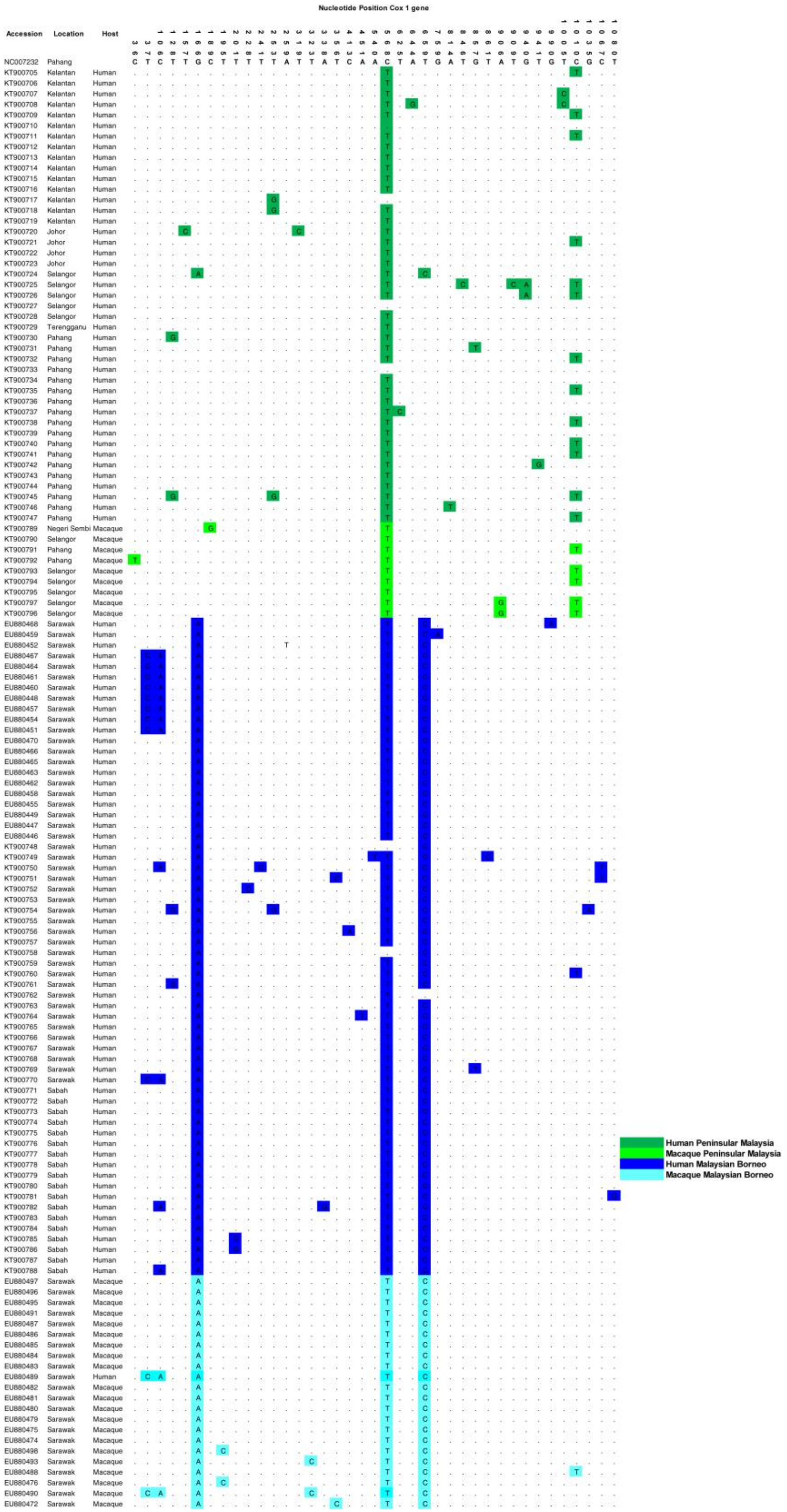

Technical Appendix Figure. Nucleotide polymorphism in the cytochrome oxidase 1 (*cox1*) gene of *Plasmodium knowlesi* isolates from humans and macaques, Peninsular Malaysia and Malaysian Borneo.
